# Real-Life Experience of mTOR Inhibitors in Liver Transplant Recipients in a Region Where Living Donation Is Predominant

**DOI:** 10.3389/fphar.2021.685176

**Published:** 2021-07-13

**Authors:** Pil Soo Sung, Ji Won Han, Changho Seo, Joseph Ahn, Soon Kyu Lee, Hee Chul Nam, Ho Joong Choi, Young Kyoung You, Jeong Won Jang, Jong Young Choi, Seung Kew Yoon

**Affiliations:** ^1^ Division of Gastroenterology and Hepatology, Department of Internal Medicine, College of Medicine, Seoul St. Mary’s Hospital, The Catholic University of Korea, Seoul, Korea; ^2^ The Catholic Liver Research Center, College of Medicine, The Catholic University of Korea, Seoul, Korea; ^3^ Department of Surgery, College of Medicine, Seoul St. Mary’s Hospital, The Catholic University of Korea, Seoul, Korea

**Keywords:** mTOR inhibitor, liver transplantation, renal dysfunction, living donation, calcineurin inhibitor

## Abstract

**Background:** Mammalian target of rapamycin (mTOR) inhibitors, such as everolimus and sirolimus, may be efficacious in preserving renal function in liver transplantation (LT) recipients while preventing hepatocellular carcinoma (HCC) recurrence.

**Materials and Methods:** In this study, we retrospectively evaluated the safety, efficacy, and renoprotective effects of mTOR inhibitors in LT recipients. Among the 84 patients enrolled, mTOR inhibitor was commenced during the first year after LT. Renal function was measured by estimated glomerular filtration rate (eGFR) using the Modification of Diet in Renal Disease equation.

**Results:** Regarding the type of mTOR inhibitor, everolimus was used in 71 patients and sirolimus in 13 patients. Concomitant tacrolimus was used in 63 patients (75.0%). For total enrolled patients, kidney function did not significantly change during 12 months after initiation of mTOR inhibitors, although tacrolimus-withdrawn patients (*n* = 21) showed better kidney function compared to tacrolimus-minimized patients (*n* = 63) after conversion. However, a significant improvement in kidney function was observed in the eGFR <60 ml/min/1.73 m2 group (*n* = 19) 12 months after initiation of mTOR inhibitors, for both patient groups with early + mid starters (*n* = 7, stating within 1 year after LT) and late starters (*n* = 12, starting over 1 year after LT). mTOR inhibitors were safely administered without serious adverse events that led to drug discontinuation.

**Conclusion:** We demonstrated that patients with renal impairment showed significant improvement in renal function regardless of the timing of mTOR inhibitor start, suggesting that switch to mTOR inhibitors may be beneficial when renal function declines.

## Introduction

The liver is considered an immune-tolerant organ. It has been reported that the liver contains tolerance mechanisms to prevent immune hyper-activation in response to foreign antigens (Sung and Jang, 2018). Graft survival rates after liver transplantation (LT) have improved to 90% at the 1 year and 75% by the fifth year (Saliba et al., 2019). Recently, however, LT recipients have become older and possess various comorbidities (Sho et al., 2020). Currently, two principal calcineurin inhibitors (CNIs) used for immunosuppressive treatment after LT are tacrolimus (TAC) and cyclosporin, and they have been emphasized as the etiology of acute and chronic kidney disease (CKD) in LT patients. CKD has an annual incidence of 8%, affecting 18% of patients 5 years after LT (Nogueras Lopez et al., 2020). CKD can be categorized in relation to the estimated glomerular filtration rate (eGFR) using the Modification of Diet in Renal Disease (MDRD) equation (<60 ml/min/1.73 m^2^) (Bilbao et al., 2015). After CKD develops, frequently prescribed alternatives to TAC include anti-metabolites, such as mycophenolic acid derivatives, and mammalian target of rapamycin (mTOR) inhibitors (Saliba et al., 2011). Among these drugs, mTOR inhibitors have been shown to have a favorable adverse event profile and to be effective in preventing acute cellular rejection while protecting kidney function in LT patients to whom immunosuppressants are usually administered in the outpatient clinic (De Simone et al., 2009; Saliba et al., 2011; De Simone et al., 2012; Bilbao et al., 2015; Saliba et al., 2017; Saliba et al., 2019; Nogueras Lopez et al., 2020; Saliba et al., 2020).

A recent observational CERTITUDE study (Saliba et al., 2019) prospectively observed and analyzed data from LT recipients who completed the SIMCER trial (Saliba et al., 2017); both studies showed renoprotective benefits of mTOR inhibitors. At 1 month after transplantation, SIMCER compared patients receiving everolimus with stepwise TAC withdrawal to those receiving standard TAC. Introduction of everolimus with gradual TAC withdrawal after LT was associated with significantly better kidney function than TAC-only immunosuppression (Saliba et al., 2017). 6 months after LT, SIMCER trial was finished and 65 everolimus-treated and 78 TAC-treated patients continued CERTITUDE trial. The mean eGFR was significantly higher with everolimus than with TAC from the third to 12th month after LT (Saliba et al., 2019). In addition, the H2307 trial showed that the early (1 month after LT) introduction of everolimus with reduced TAC regimen had similar efficacy with better renoprotective effect at 24 months after LT (Jeng et al., 2018; Lee et al., 2021). However, the rate of stopping mTOR inhibitors due to the adverse events was relatively high, affecting approximately one quarter of patients (Nashan, 2018). Moreover, for maintenance patients (>1 year after LT), data regarding mTOR inhibitor-based immunosuppression are limited, although previous reports showed that late conversion to mTOR inhibitors (>1 year) appeared ineffective in renal protection (Nashan, 2018).

Compared to other surgical or ablative procedures, LT is accountable for a 10 years recurrence-free survival rate of more than 70% in hepatocellular carcinoma (HCC) cases (Sung et al., 2017; Rodriguez-Peralvarez et al., 2018; Sho et al., 2020). On the other hand, tumors recur in approximately 20% of patients who undergo LT, and this proportion is even higher in cases with over-Milan criteria or with pathological microvascular invasion (Sung et al., 2017; Yang et al., 2019; Nishida, 2020). The likelihood of HCC recurrence can be diminished by tapering immunosuppressants (Vivarelli et al., 2008; Dumortier et al., 2016). However, this approach is related to a possible rejection risk, which can be overcome in clinical practice by using CNIs alongside other immunosuppressants, particularly mTOR inhibitors, such as everolimus or sirolimus. mTOR inhibitors have considerable appeal in HCC patients undergoing LT, as they may prevent the proliferation of cancer cells (Jung et al., 2018). Recent evidence has shown that the mTOR pathway is expressed at a higher level in HCC cells, and tumor regression was achieved in HCC-harboring animals by treatment with rapamycin (Lee et al., 2020a; Chao et al., 2020; Ferrin et al., 2020). Previous studies demonstrated that mTOR inhibitors may reduce the recurrence of HCC after LT (Grigg et al., 2019), although most of these studies compared an mTOR inhibitor plus a low dose of TAC and a regular dose of TAC only. Another recent prospective study that evaluated the anti-recurrence effects of early everolimus use after LT did not show significant results (Rodriguez-Peralvarez et al., 2018).

In this study, we retrospectively evaluated the safety, efficacy, and renoprotective effects of mTOR inhibitors in liver transplant recipients with HCC. This is the first real-life report of mTOR inhibitor experience in Korea, where hepatitis B virus (HBV) infection is a principal cause of HCC and living donor LT (LDLT) is predominant.

## Materials and Methods

### Study Design and Population

**FIGURE 1:**
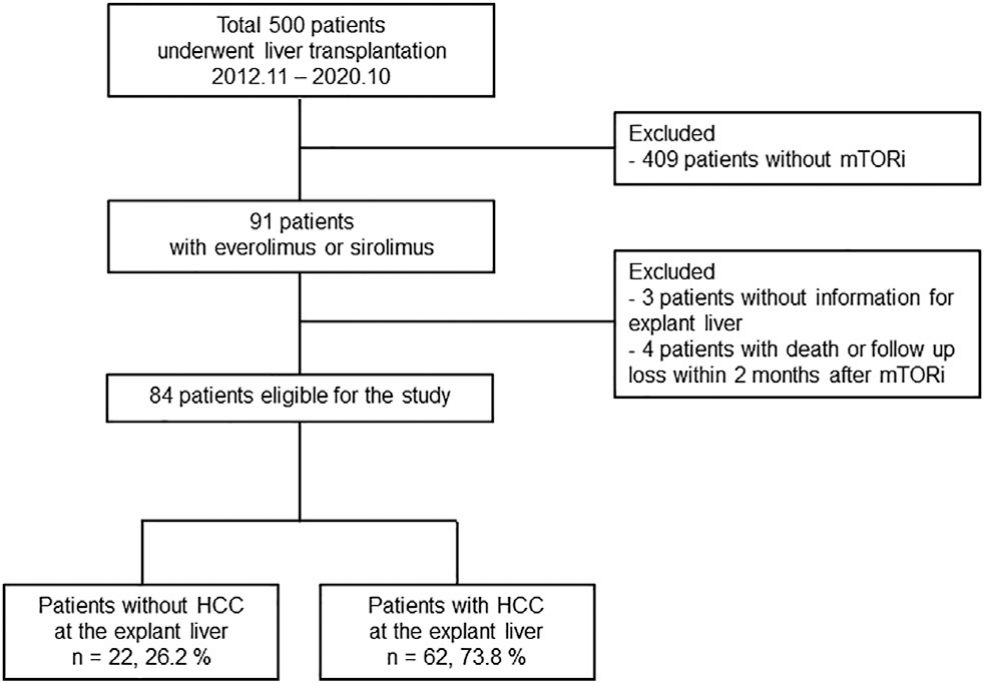
Study population.

The study population is shown in Figure 1. The present study was approved by the Institutional Review Board of Seoul St. Mary’s Hospital (KC20RISI0973). Seoul St. Mary’s Hospital (Seoul, Korea) is one of the largest liver transplantation centers in Korea, where HBV infection is endemic and LDLT for HCC patients predominates. This retrospective observational study initially screened 500 patients who underwent LT at Seoul St. Mary’s Hospital between November 2012 and October 2020 (Figure 1). Among them, 91 patients received everolimus or sirolimus as immunosuppressants. After excluding three patients without information for explant liver and four patients with short-term use of mTORi (<2 months), 84 patients were finally enrolled in the subsequent analyses. These patients were evaluated with respect to the utilization of mTOR inhibitors in association with TAC minimization or discontinuation and the median observational period was 1,016 days. Among these, 62 LT recipients had HCC before LT, which was diagnosed by at least two separate dynamic radiological studies (including computed tomography [CT], magnetic resonance imaging [MRI], or hepatic angiography) or liver biopsy.

### Surgical Procedures and Postoperative Surveillance of HCC

The surgical procedure of LT has been described previously (Sung et al., 2017). The number of LDLT has increased significantly because donors have not been sufficiently available for deceased-donor LT in Korea (Kim et al., 2012). Blood tests, including tumor markers such as alpha-fetoprotein and des-gamma carboxyprothrombin, were performed monthly during the 1^st^ year after LT. Liver dynamic CT/MRI using liver-specific contrast agents (Primovist) was performed every 3 months during the 1^st^ year, every 6 months during the 2^nd^ year, and every 1 year thereafter. If recurrence of HCC was suspicious, additional studies such as fMRI or positron emission tomography–computed tomography (PET-CT) was performed as previously described (Yang et al., 2019).

### Immunosuppression

The following drugs were used as immunosuppressants at baseline as previously described (Na et al., 2014): TAC, prednisolone, and mycophenolate mofetil (MMF). Briefly, TAC level was maintained at 7–10 ng/ml for the 1st month after LT and at 5–7 ng/ml following that, unless they were tapered or stopped due to the use of mTOR inhibitors. Prednisolone was tapered out 1 month after LT, and MMF was stopped 6 months after LT. The main reason for the start of mTOR inhibitors was recurred HCC before the start of mTOR inhibitors (27.4%, 23/84).

### Evaluation of Biopsy-Proven Acute Rejection (BPAR)

Liver biopsy was performed to confirm cellular rejection. Biopsy specimens were examined by pathologists using the Banff scheme (1997). Portal, bile duct, and venous inflammation were graded (0, absent, to 3, severe). From these scores, the overall rejection activity index (RAI) was calculated, and BPAR was defined as RAI ≥ 3.

### Measurement of Renal Function

Renal function was measured at the start of mTOR inhibitors and after 1, 3, 6, and 12 months. Glomerular filtration rate (GFR) was calculated as previously described by the MDRD study group (Levey et al., 1999). The formula is as follows: GFR, in mL/min/1.73 m^2^ = 175 × SCr [exp (−1.154)] × age [exp (−0.203)] × (0.742 if female) × (1.21 if black). The definition of CKD was reduced renal function for ≥3 months. The stage of CKD was defined according to the GFR as previously described (Webster et al., 2017) (Stage 1, GFR ≥90 ml/min/1.73 m^2^; Stage 2, GFR 60–89 ml/min/1.73 m^2^; Stage 3, GFR 30–59 ml/min/1.73 m^2^; Stage 4, GFR 15–29 ml/min/1.73 m^2^; Stage 5, GFR < 15 ml/min/1.73 m^2^).

### Statistical Analysis

Continuous variables were showed as median with interquartile range (IQR) and categorical variables were showed as numbers (%). Continuous variables between groups were compared using the Mann-Whitney U tests (for unpaired groups) or Wilcoxon tests (for paired groups). Statistical analyses were performed using R software (http://cran.r-project.org/) and GraphPad Prism version 6.0 (GraphPad Software, United States). Statistical significance was defined as a *p*-value < 0.05.

## Results

### Patient Demographics

Table 1 shows the demographic data and baseline characteristics of all enrolled patients (everolimus + sirolimus) and patients with everolimus use (everolimus group). In total patients, HCC resulting from chronic hepatitis B (*n* = 54, 64.3%) was the most common reason for LT. A total of 72 patients (85.7%) underwent LDLT, and 14 patients (16.7%) underwent ABO-incompatible LT. The median model for end-stage liver disease (MELD) score was 9.4. Most patients (*n* = 72, 85.7%) received basiliximab induction therapy. Initially, TAC, prednisolone, and MMF are usually administered to LT recipients. 6 months after LT, most patients (*n* = 78, 92.9%) still received TAC. mTOR inhibitor was commenced following median of 269 days after LT. A total of 50 patients (59.5%) received mTOR inhibitors within 12 months after LT. The mean duration of mTOR inhibitor use was 420.58 days for early + mid starter (mTOR inhibitor start: 0–12 months after LT) and 436.3 days for late starter (mTOR inhibitor start: > 12 months after LT).

**TABLE 1:** Patient demographics.

Variables	All patients, *n* = 84 (sirolimus + everolimus)	Patients with everolimus, *n* = 71
Male gender	70 (83.3)	59 (83.1)
Age, years	55.7 ± 8.6	55.5 ± 8.8
Underlying liver disease		
HBV	54 (64.3)	43 (60.6)
HCV	6 (7.1)	4 (5.6)
Alcohol	15 (17.9)	15 (21.1)
Others	9 (10.7)	9 (12.7)
HCC at explant liver	62 (73.8)	49 (69.0)
LDLT	72 (85.7)	60 (84.5)
ABO incompatible LT	14 (16.7)	12 (16.9)
Laboratory findings at the time of LT		
Creatinine, mg/dL	0.8 (0.7–1.0)	0.9 (0.7–1.0)
GFR, mL/min	90.9 (77.8–104.0)	90.0 (70.5–103.0)
Total bilirubin, mg/dL	1.5 (0.7–4.0)	1.8 (0.7–4.5)
INR	1.3 (1.2–1.7)	1.3 (1.2–1.8)
MELD	9.4 (4.3–16.7)	10.1 (5.0–17.3)
Basiliximab induction	72 (85.7)	61 (85.9)
Types of immunosuppressants 6 months after LT		
Tacrolimus	78 (92.9)	69 (97.2)
mTOR inhibitor	23 (27.4)	18 (25.4)
Steroids	8 (9.5)	8 (11.3)
MMF	35 (41.7)	29 (40.8)
Time to conversion to mTOR inhibitor, months		
0–3 (early), median	10 (11.9)	9 (12.7)
4–12 (mid), median	40 (47.6)	31 (43.7)
>12 (late), median	34 (40.5)	31 (43.7)
Duration of mTOR inhibitor maintenance, days	344.0 (146.5–528.5)	449 (162.5–549.5)
Reason for mTOR inhibitor start		
Impaired renal function	17 (20.2)	16 (22.5)
Non-hepatic *de novo* cancers	7 (8.3)	7 (9.9)
Prevention of HCC recurrence	22 (26.2)	22 (31.0)
Recurred HCC	23 (27.4)	11 (15.5)
Intolerance to other immunosuppressants	10 (11.9)	10 (14.1)
Others	5 (6.0)	5 (7.0)
Types of mTOR inhibitor		
Everolimus	71 (84.5)	71 (100.0)
Sirolimus	13 (15.5)	0 (0)
CKD		
Stage 1, 2	61 (72.6)	48 (67.6)
Stage 3	16 (19.0)	16 (22.5)
Stage 4	5 (6.0)	5 (7.0)
Stage 5	2 (2.4)	2 (2.8)
Concomitant immunosuppressants with mTOR inhibitor		
Tacrolimus	63 (75.0)	60 (84.5)
Steroids	6 (7.1)	6 (8.5)
MMF	17 (20.2)	13 (18.3)
BPAR after mTOR inhibitor	8 (9.5)	7 (9.9)
Death	22 (26.2)	15 (21.1)
Cause of death		
HCC recurrence	11 (55.0)	6 (40.0)
Liver failure	4 (20.0)	4 (26.7)
Infection	4 (20.0)	4 (26.7)
Other	1 (5.0)	1 (6.7)

Data are given as n (%) or median (IQR). HBV, hepatitis B virus; HCV, hepatitis C virus; HCC, hepatocellular carcinoma; LDLT, living donor liver transplantation; LT, liver transplantation; GFR, glomerular filtration rate; INR, international normalized ratio; MELD, model for end-stage liver disease; MMF, mycophenolate mofetil; CKD, chronic kidney disease; BPAR, biopsy proven acute rejection; HCC, hepatocellular carcinoma.

The reasons for mTOR inhibitor initiation were as follows (in all patients): impaired renal function (*n* = 17, 20.2%), non-hepatic *de novo* cancers (*n* = 7, 8.3%), prevention of HCC recurrence (*n* = 22, 26.2%), presence of HCC recurrence (*n* = 23, 27.4%), intolerance to other immunosuppressants (*n* = 10, 11.9%), and others (*n* = 5, 6.0%). Non-hepatic *de novo* cancers were as follows: one bladder cancer patient, two head and neck cancer patients, one lymphoma patient, one kidney cancer, one colon cancer, and one esophageal cancer. TAC was concomitantly administered with mTOR inhibitors in 63 patients (75.0%) and discontinued in 21 patients (25.0%). Regarding types of mTOR inhibitors, everolimus was used in 71 patients and sirolimus was used in 13 patients. We also summarized same clinical variables in everolimus group, and obtained similar pattern of results (Table 1).

The baseline median eGFR was 78.5 ml/min, and only one of the 84 patients was receiving renal replacement therapy following surgery when mTOR inhibitors were commenced. Renal transplantation was not required for any patients. A total of 61 (72.6%) patients had CKD stage 1 or 2, whereas 23 (27.4%) patients had CKD stage 3 or higher. BPAR following LT occurred in 8 (9.5%) patients with TAC withdrawal or minimization. The rejection rates between TAC withdrawn and TAC minimizing groups were not different (2/21 = 9.5% vs. 6/63 = 9.5%). Death occurred in 22 patients (26.2%) during the surveillance period. Causes of death were recorded as HCC recurrence (*n* = 11, 55.0%), liver failure (*n* = 4, 20.0%), and infection (*n* = 4, 20.0%). No deaths were attributed to mTOR inhibitors.

**FIGURE 2:**
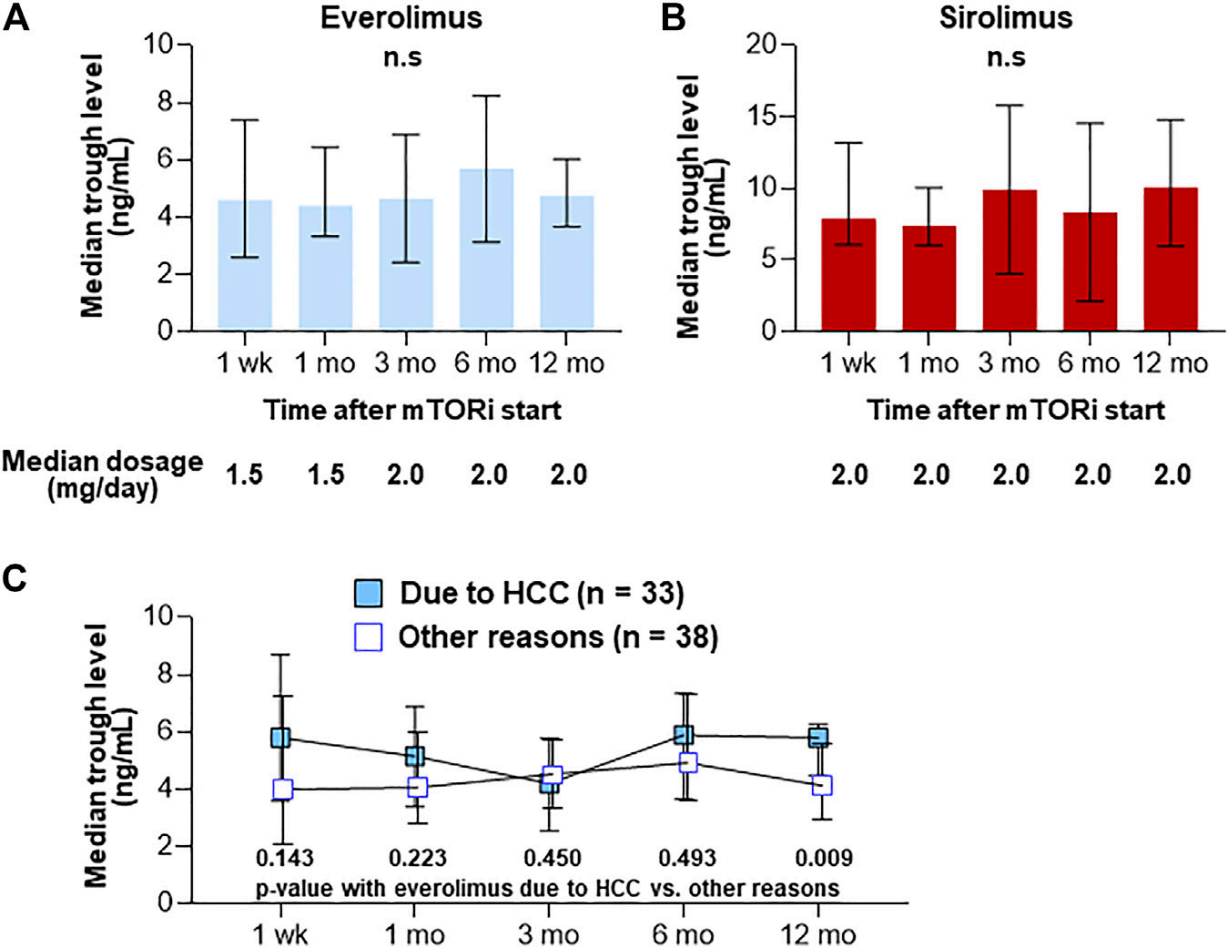
Trough levels after the introduction of everolimus (*n* = 71) **(A)** and sirolimus (*n* = 13) **(B)**. Trough levels after the introduction in patients who received everolimus to prevent or to treat HCC recurrence (*n* = 33) to the other group (*n* = 38) **(C)**. Graphs show median with IQR trough levels of each agent. n.s., not significant.

After the start of mTOR inhibitors, their trough levels were measured at week 1, month 1, month 3, month 6, and month 12 (Figures 2A,B). The median initial dose of everolimus was 1.5 mg/day (range, 1–3 mg/day) and then its dose was adjusted to achieve a target trough level of 3–8 ng/ml. Median trough levels of everolimus were 4.6, 4.4, 4.2, 5.2, and 4.8 ng/ml, respectively. The initial dose of sirolimus was 2 mg/day (range, 1–4 mg/day) and then its dose was adjusted to achieve a target trough level of 4–20 ng/ml. Median trough levels of sirolimus were 7.9, 7.4, 7.9, 6.9, and 10.1 ng/ml, respectively. When comparing trough levels of patients who received everolimus to prevent or to treat HCC recurrence (*n* = 33) to the other group (*n* = 38) at each time-point, the trough level at month 12 after LT in HCC group was significantly higher (median 5.8 vs. 4.2 ng/ml, *p* = 0.009) (Figure 2C).

### Adverse Events Following the Introduction of mTOR Inhibitors

Table 2 shows the adverse events within 2 weeks after the start of mTOR inhibitors. mTOR inhibitors was not discontinued in any of the patients, although six patients (7.1%) developed thrombocytopenia after mTOR inhibitor use. Proteinuria (*n* = 10, 11.9%), mouth ulceration (*n* = 5, 6.0%), and GI trouble (*n* = 8, 9.5%) were also common adverse events associated with mTOR inhibitors.

**TABLE 2:** Adverse events by mTOR inhibitor use (within 2 months).

Variables	All patients, *n* = 84 (sirolimus + everolimus)	Patients with everolimus, *n* = 71
Anemia	3 (3.6)	2 (2.8)
Leukopenia	5 (6.0)	5 (7.0)
Thrombocytopenia	6 (7.1)	5 (7.0)
Aminotransferase elevation	7 (8.3)	5 (7.0)
Proteinuria	10 (11.9)	8 (11.3)
Mouth ulceration	5 (6.0)	3 (4.2)
GI trouble	8 (9.5)	5 (7.0)

Data are given as n (%). GI, gastrointestinal.

### Demographics of the Patients Who had HCC Before LT

Table 3 shows the demographics of the patients who had HCC before LT and administered mTOR inhibitors (*n* = 62, 73.8%). Explant pathology demonstrated that 22 patients (33.5%) were considered outside the Milan criteria. Thirty-nine patients (62.9%) started mTOR inhibitors before HCC recurrence. For treatment of recurrent HCC, systemic chemotherapy, including sorafenib or lenvatinib, was primarily used (59.3%).

**TABLE 3:** Demographics of the patients who had HCC before LT.

Patients with HCC before LT (n = 62, 73.8%)	
**Explant pathology**	
HCC number	
Single	21 (33.9)
Multiple	41 (66.1)
HCC maximal diameter (cm)	3.5 (2.5–4.7)
Pathological over-Milan	22 (35.5)
Microvascular invasion	27 (43.5)
**Timing of mTORi start**	
before HCC recurrence	39 (62.9)
after HCC recurrence	23 (37.1)
**Treatment for recurrent HCC**	
Systemic chemotherapy	16 (59.3)
Transarterial chemoembolization	6 (22.2)
Surgery (including metastasectomy)	5 (18.5)

Data are given as n (%) or median (IQR). HCC, hepatocellular carcinoma; LT, liver transplantation; min, minimal.

### Renal Function After mTOR Inhibitor Introduction in the Whole Population by eGFR (MDRD)

**FIGURE 3:**
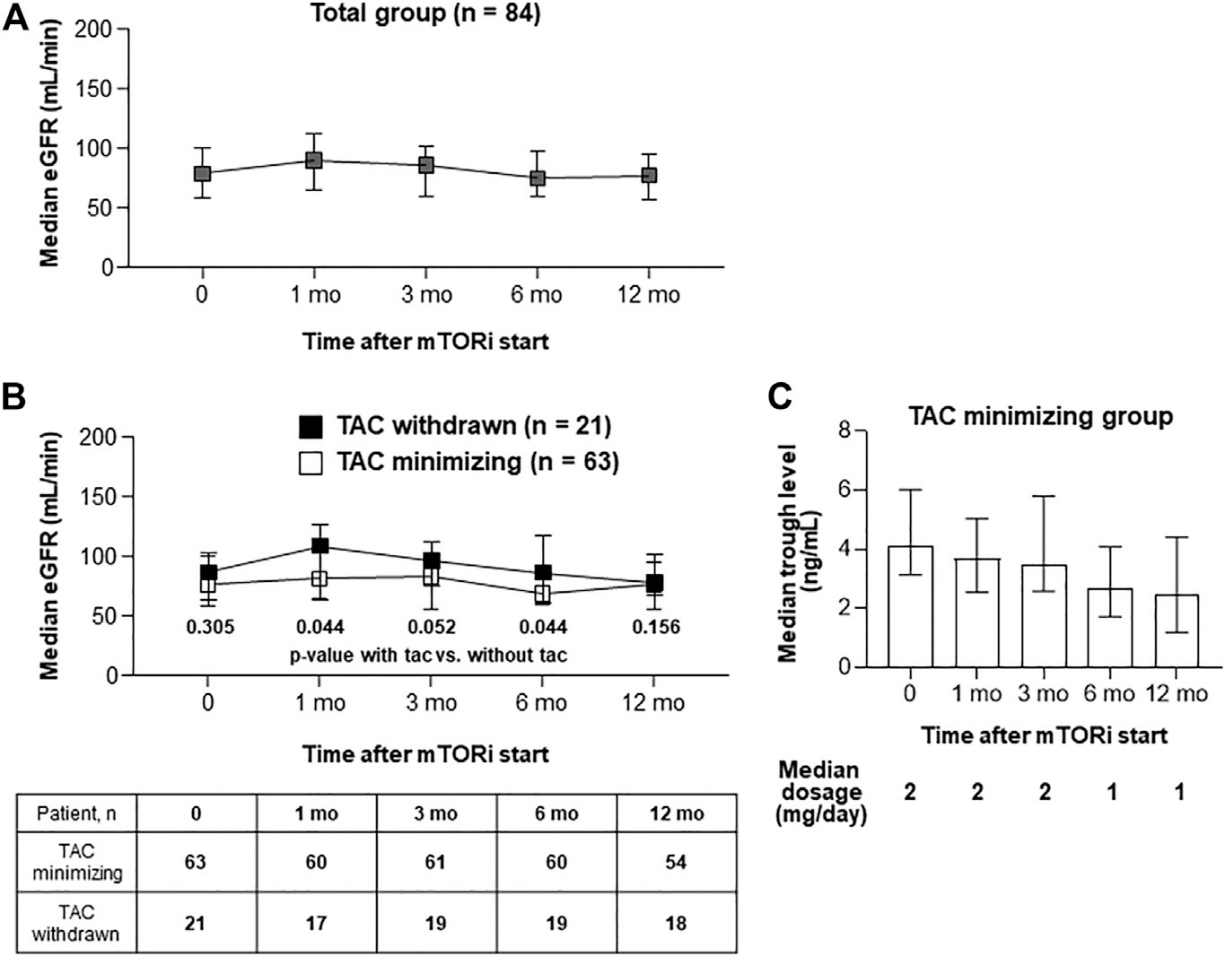
Serial renal functions after the introduction of mTOR inhibitors. **(A, B)** Graphs show median with IQR of GFR level in each group Serial GFR in total patients **(A)**. Serial GFR in patients with TAC (*n* = 63) or without TAC (*n* = 21). Comparing GFR levels between TAC withdrawn and with TAC minimizing groups, Mann-Whitney test was used **(B)**. **(C)** A graph that shows (1) median dosage of TACs after mTOR inhibitors start, and (2) median with IQR of tacrolimus trough levels at each time-point.

In the whole enrolled subjects, renal function (assessed as eGFR using the MDRD formula) did not significantly change up to 12 months after conversion (Figure 3A). The median eGFR values at 1, 3, 6, and 12 months after conversion to mTOR inhibitors were 90.0, 75.5, 74.5, and 76.8 ml/min, respectively (Figure 3A). TAC treatment was minimized (75%) or aborted (25.0%) in patients given mTOR inhibitors. In patients from whom TAC was withdrawn, mean eGFR values at 1, 3, 6, and 12 months after conversion to mTOR inhibitors were 110.0, 98.0, 87.5, and 82.0 ml/min, respectively (Figure 3B). The latter group (TAC withdrawn) exhibited significantly higher eGFR than the former group (TAC minimized) at 1 and 6 months after conversion to mTOR inhibitors (Figure 3B). For TAC minimizing group, after mTOR inhibitors were started, TAC dosage and trough levels remained very low (Figure 3C).

### Renal Function After mTOR Inhibitor Introduction in the Patients With CKD by eGFR (MDRD)

**FIGURE 4:**
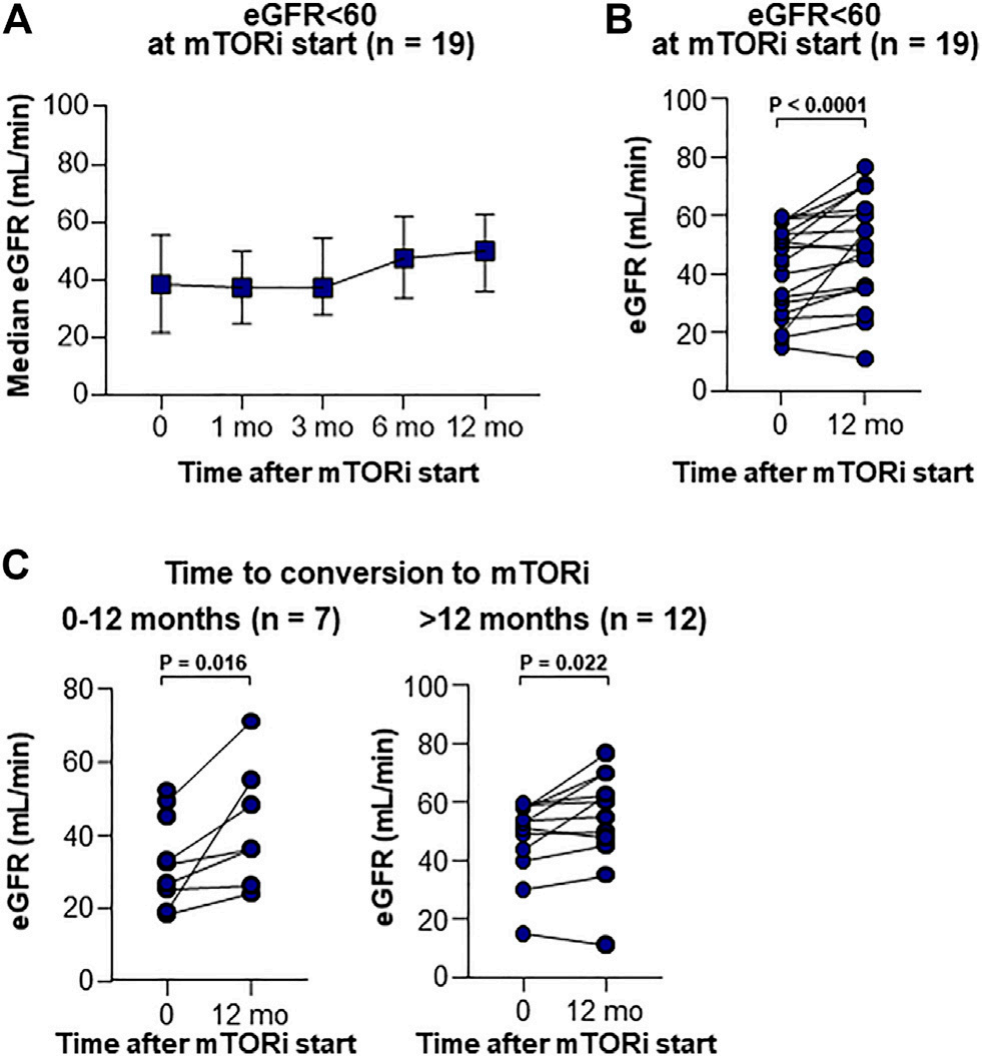
Serial renal functions in CKD patients after the introduction of mTOR inhibitors. **(A)** Serial median with IQR of GFR level in patients <60 ml/min. **(B)** Changes of GFR in CKD patients between at the start and at 12 months after the introduction of mTOR inhibitors. Wilcoxon’s test was used for comparing GFR between baseline and 12 months after mTOR inhibitor start. **(C)** Changes of GFR in CKD patients between at the start and at 12 months after the introduction of mTOR inhibitors according to the time from LT to mTOR inhibitor start. Wilcoxon’s test was used for comparing GFR between baseline and 12 months after mTOR inhibitor start.

Renal function was also analyzed according to the presence or absence of CKD, as measured by eGFR (MDRD) < 60 (*n* = 19) or ≥60 ml/min/1.73 m^2^ (*n* = 65) at the start of mTOR inhibitors; a significant improvement in renal function was noted in the CKD group after 12 months (Figures 4A,B). This improvement of renal function was also confirmed when patients were grouped into early + mid starter (mTOR inhibitor start: 0–12 months after LT, Figure 4C) and into late starter (mTOR inhibitor start: > 12 months after LT, Figure 4C).

## Discussion

The aim of this study was to evaluate the safety, efficacy, and renoprotective effects of mTOR inhibitors in liver transplant recipients with HCC. This is the first real-life report of mTOR inhibitor application in Korea, where HBV infection is the principal cause of HCC and LDLT is predominant. A considerable number of the patients in our study received mTOR inhibitors 3 months after transplantation (88%), and 40% of the patients started mTOR inhibitors over 12 months after transplantation, suggesting that our data may reflect the effects of mTOR inhibitors in the maintenance phase after LT.

The patient prognosis following LT has been transformed by the use of immunosuppressive agents and improvements in perioperative care (Kang et al., 2018). However, enhanced long-term survival has been accompanied by an increase in the prevalence of late complications following LT, including CKD (Lin et al., 2017). The H2304 trial demonstrated that starting everolimus 1 month after transplant with reduced TAC dose achieved an improvement in renal function compared to the standard TAC-based therapy. A recent meta-analysis that included four randomized clinical trials of primary adult LT recipients with baseline GFR >30 ml/min who received everolimus with CNI minimization or withdrawal demonstrated that everolimus use with CNI minimization in LT recipients is associated with improved renal function at 12 months. In the current study, renal impairment was also one of the major rationales for commencing mTOR inhibitors. Renal function was analyzed according to the presence or absence of CKD at the time of mTOR inhibitor initiation, and a significant improvement in renal function was noted in the CKD group after 12 months of mTOR inhibitor use. Recent reports have demonstrated that earlier conversion to mTOR inhibitors is associated with better recovery of renal function in patients (Yanik et al., 2016; Saliba et al., 2020). However, in our data, patients with late conversion (>1 year) also showed significant improvement in renal function, suggesting that changes to mTOR inhibitors are required when renal function declines, regardless of the duration after LT.

In our study, there were 8 cases (9.5%) of BPAR after mTOR inhibitors start. Bilbao et al. reported an incidence of acute rejection in 14.9% of cases after conversion (Bilbao et al., 2015). The rejection rate in our cohort was low because 75% of patients maintained a low dose of TAC after mTOR inhibitor initiation and 20% of patients maintained a low dose of MMF. In the current population, the adverse event profile was similar with that reported in other mTOR inhibitor-treated populations (Chapman et al., 2017). In a recent observational CERTITUDE study (Saliba et al., 2019), the rate of stopping mTOR inhibitors due to the adverse events was relatively high, affecting approximately one quarter of patients (Nashan, 2018). In our study, the everolimus trough concentrations did not exceed the recommended levels for LT recipients. They were even closer to the inferior threshold at follow-up, causing none of the patients stopped mTOR inhibitors due to the adverse effects.

Much controversy continues to surround the function of mTOR inhibitors in post-LT HCC recurrence prevention. As previously mentioned, a recent meta-analysis demonstrated that early mTOR-inhibitor-based immunosuppression improves recurrence-free survival over 3 years after LT and reduces the recurrence rate compared to the standard CNI-based immunosuppression, with no significant increase in the frequency of graft rejection (Grigg et al., 2019). In addition, H2304 trial showed that HCC recurrence was only observed in patients with TAC only regimen, but not in patients with everolimus with reduced TAC regimen at 12 months after LT (Jeng et al., 2018). Furthermore, at 24 months after LT, early introduction of everolimus with reduced TAC regimen was associated with the lower HCC recurrence in HCC patients beyond Milan (Lee et al., 2021). Indeed, immunosuppression strategies underpinned by mTOR inhibitors have been introduced in numerous institutions as standard clinical practice for HCC-transplanted cases, despite the lack of evidentiary scientific support (De Simone et al., 2017). Moreover, two recent multicenter prospective trials (one performed in Europe (Geissler et al., 2016) and the other performed in Korea (Lee K. W. et al., 2020) that compared mTOR inhibitor-only and TAC-only regimens in terms of the recurrence of HCC showed conflicting results for recurrence-free survival and overall survival after LT. Future prospective trials with a larger number of enrolled patients are needed to confirm the efficacy of mTOR inhibitors in preventing the recurrence of HCC.

The limitations of our study are as follows: retrospective study in a single hospital, a small number of analyzed patients, heterogeneity of the cohort, and relatively late conversion to mTOR inhibitors.

In conclusion, this study demonstrated the safety, efficacy, and renoprotective effects of mTOR inhibitors in LT recipients with HCC. This is the first real-life report of mTOR inhibitor application in Korea, where HBV infection is the principal cause of HCC and LDLT is predominant. Future prospective studies comprising patients with or without mTOR inhibitors will elucidate the blood levels and the role of mTOR inhibitors in rescuing renal function and prolonging recurrence-free survival and overall survival in LT patients.

## Data Availability

The original contributions presented in the study are included in the article/Supplementary Material, further inquiries can be directed to the corresponding author.
